# Assessing the Education and Training Needs of Nebraska’s Public Health Workforce

**DOI:** 10.3389/fpubh.2015.00161

**Published:** 2015-06-22

**Authors:** Brandon L. Grimm, Patrik Johansson, Preethy Nayar, Bettye A. Apenteng, Samuel Opoku, Anh Nguyen

**Affiliations:** ^1^College of Public Health, University of Nebraska Medical Center, Omaha, NE, USA; ^2^Jiann-Ping Hsu College of Public Health Georgia Southern University, Statesboro, GA, USA; ^3^Nebraska Health Network, Omaha, NE, USA

**Keywords:** public health competencies, needs assessment, workforce training needs, education and training, public health administration

## Abstract

**Introduction:**

In 2012, the Great Plains Public Health Training Center (Grant #UB6HP22821) conducted an online survey of state and local health departments and the American Indian (tribal clinics, tribal health departments, and urban Indian clinic) public health workforce across three professional levels. The objectives of the needs assessment were to determine the competency levels of the state’s public health workforce, assess gaps in public health competencies, identify public health training interests, needs, and preferences, and to determine the barriers and motivators to participate in public health training.

**Methods:**

The assessment was developed using the Council on Linkages Between Academia and Public Health Practice, Core Competencies for Public Health Professionals survey (1). The final assessment was created and piloted by numerous individuals representing practice and academia.

**Results:**

Respondents identified cultural competency and communication skills as the two most important public health competency domains. Although the public health professionals perceived that they were least proficient in the area of policy development and program planning, participants identified the greatest needs for training in financial planning and management skills and analytical/assessment skills. In general, respondents preferred instructor-led interactive training sessions offered as onsite multi-day workshops or computer-based courses. Respondents identified obesity, health disparities, physical activity, chronic diseases, and diabetes as the top five public health topical areas.

**Conclusion:**

These priorities align with State and National public health plans. The findings of the needs assessment were used to tailor educational opportunities to build the capacity of Nebraska’s public health system. Additionally, the results were used to develop workforce development plans for numerous local health departments throughout Nebraska.

## Introduction

Over more than two decades, the Institute of Medicine has reiterated the strong recommendation that public health professionals possess the appropriate education and training to perform the required roles by this workforce (2, 3). Most recently, Healthy People 2020 has established the newest related national goal: *ensure that Federal, State, Tribal, and local health agencies have the necessary infrastructure to effectively provide essential public health services*. Specifically, Healthy People 2020 states that this infrastructure requires (1) a capable and qualified workforce; (2) up-to-date data and information systems; and (3) public health agencies capable of assessing and responding to public health needs (4).

With the aging of the public health workforce, experts fear that the number of trained workers available will be insufficient to replace the number retiring in the next decade. In 2012, approximately 110,000 workers, nearly 25% of the current workforce, were eligible to retire (5). By 2020, the Association of Schools and Programs of Public Health (ASPPH) reports, the U.S. will face a shortage of 250,000 public health workers. Recent workforce surveys by the Association of State and Territorial Health Officials (ASTHO) and the National Association of County and City Health Officials (NACCHO) support these claims. The average age of a state public health worker was 47, and the average age of a new hire in state government was 40 years. The average age of local health departments’ top executives, meanwhile, was 53 years. In 2012, over 50% of some state health agency workforces were eligible to retire (6, 7). According to ASPPH, in 2008, schools of public health would need to train three times the current number of graduates to meet projected shortfalls. Given this challenge and the need for formal training offerings, providing lifelong learning through opportunities such as short courses, certificate programs, and distance learning has been recommended, in addition to encouraging governmental health agencies at all levels to develop succession plans that will sustain leadership (5).

These assertions hold especially true in Nebraska. Nebraska’s public health system has undergone a remarkable transformation in the last decade. Prior to the 2001 passage of Nebraska Legislative Bill 692 that established 16 new multi-county agencies, only 22 of the state’s 93 counties were served by a local public health department. By 2010, there were 23 county/regional health departments, four tribal, and one state health department that serve every county in the state. The vast majority of directors of the new departments were first-time leaders of a health agency. Only 15% indicated previous experience as a health department director and only two had a bachelor’s or master’s degree in public health. Additionally, excluding health department directors, among the estimated 600 employes in local public health agencies, only about 4% of them had completed formal public health training or certification (8).

Given the important role public health workforce plays in ensuring the health of the populace, it is vital that they have received adequate training and education to effectively carry out their functions. To strengthen the education and training of US public health professionals, in 1999, the Health Resources and Services Administration (HRSA), beginning with two pilot sites, established the Public Health Training Center (PHTC) program (9). HRSA presently supports ten regional PHTCs across the US.

The Great Plains PHTC was established in 2011with funding from HRSA. The goals of the Great Plains PHTC were to (1) Establish an effective, durable infrastructure to sustain the Great Plains PHTC, (2) Conduct comprehensive assessments of workforce development and training needs, and (3) Increase and strengthen the technical, scientific, managerial, and leadership capacity in underserved Nebraska and Tribal communities.

The Great Plains PHTC conducted the first ever education and training needs assessment in Nebraska between September 2011 and June 2012. The objectives of the needs assessment included: (a) determining the competency levels of the state’s public health workforce in performing essential public health functions; (b) assessing gaps in public health competencies; (c) identifying public health training interests, needs, and preferences; and (d) determining the barriers and motivators to participating in public health training.

## Materials and Methods

The needs assessment used the principles of practice-based systems research. The goal of practice-based systems research is to improve quality, performance, efficiency, and effectiveness of public health systems that affect community health outcomes (10). The University of Nebraska Medical Center Institutional Review Board approved the study.

The Great Plains PHTC assessment and evaluation workgroup designed the tool. The membership of the workgroup included a geographically diverse representation of practitioners from state, local, and tribal public health departments, federally qualified health centers, and faculty and staff from numerous Universities throughout Nebraska. Needs assessment tools used by other PHTCs across the country, including the Georgia PHTC and the Northwest Center for Public Health Practice (11, 12) and a literature review also informed the design of the needs assessment. The assessment tool used the council on linkages (COL) Between Academia and Public Health Practice, Core Competencies for Public Health Workforce survey to measure competencies of the public health workforce (1). Face validity was established by having experts in the field (i.e., workforce development, assessment of competencies, survey design) and public health practitioners review the tool. The experts and practitioners provided feedback and suggestions to make the assessment as useable as possible. The COL instrument assesses the skill level of respondents in carrying out essential public health functions across the eight domains: (1) Analytical/Assessment Skills, (2) Policy Development/Program Planning Skills, (3) Communication Skills, (4) Cultural Competency Skills, (5) Community Dimensions of Practice Skills, (6) Public Health Sciences Skills, (7) Financial Planning and Management Skills, and (8) Leadership and Systems Thinking Skills.

The survey was deployed using SurveyMonkey, an online survey and data management software. Since we did not have access to all public health employes, the survey link was sent to the health directors of 28 local, state, and tribal health departments in Nebraska with a request to forward the link to all employes. The respondents were instructed to complete one of three surveys depending on their perceived professional levels: Tier One (entry level staff), Tier Two (supervisors and managers), and Tier Three (senior managers and CEOs) (1). The effective sample sizes for tier one, two, and three respondents were 68, 45, and 20, respectively.

The assessment tool included 122 questions in three sections. The first section included demographic questions and section two asked respondents three questions about each of the competencies in the eight domains. For each item, respondents were asked to rate its importance, their perceived level of proficiency, and their interest in training on a Likert scale of 1 through 5. The final section of the assessment included questions about specific topical areas of training needs, priorities (i.e., informatics, team building, health disparities, mental health, etc.), and learning culture and style.

The results from the assessment were descriptively summarized and analyzed using SAS 9.2 software. The analysis of section two of the assessment used the average domain score for all respondents and calculated a perceived importance of the domain score, a self-perceived level of proficiency score, and an interest in training score for each domain. Additionally, mean domain scores for each tier of respondents were computed separately. A need for training score was computed for each item of the survey tool by subtracting the self-perceived level of proficiency score from the perceived level of importance score. A mean need for training score was then computed for each of the eight competency domains. The Need for Training scores ranged from -4 to + 4, with a positive score indicating a need for training. A score of 0 and below indicates no need for training. The Kansas and Ohio PHTCs survey used a similar methodology to assess training health needs for their public health workforce (13, 14).

## Results

### Demographic characteristics

The directors at each health department sent the survey to 341 public health professionals in the state. Of the 208 respondents who initiated the survey (61.3%), only 133 provided responses beyond demographic and employment characteristics, which resulted in an effective response rate of 39.0%. Table 1 provides information on the demographic characteristics of survey respondents. The results are presented in aggregate and by tiers. The majority of the 208 respondents who responded to the demographic and employment sections of the survey were female (*N* = 166/208; 78.9%), White (*N* = 167/208; 80.3%), and non-Hispanic (*N* = 185/208; 88.9%). The demographic characteristics were similar across all three tiers. However, there was a higher proportion of Hispanics in tier one compared to the other two tiers (12.8 vs. 2.9 and 0.0%, respectively). In addition, compared to tiers one and three, a higher proportion of the respondents in tier two were over 60 years old (27.5 vs. 10.1 and 10.0%, respectively).

**Table 1:** **Demographic characteristics of respondents**.

	Total respondents	Tier one	Tier two	Tier three
	*N* = 208	*N* = 109	*N* = 69	*N* = 30
	*N* (%)	*N* (%)	*N* (%)	*N* (%)
**Gender**
Female	166 (78.9)	93 (85.3)	51 (73.9)	22 (73.3)
Male	41 (19.7)	15 (13.8)	18 (26.1)	8 (26.7)
Missing	1 (0.5)	1 (4.6)	0 (0.0)	0 (0.0)
**Race**
White	167 (80.3)	84 (77.1)	59 (85.5)	24 (80.0)
Black/African American	5 (2.4)	1 (0.5)	1 (1.4)	3 (10.0)
Asian or Pacific Islander	4 (1.9)	1 (0.5)	2 (2.9)	1 (3.3)
American Indian/Alaskan Native	17 (8.2)	11 (10.1)	5 (7.2)	1 (3.3)
Other	11 (5.3)	8 (7.3)	2 (2.9)	1 (3.3)
Missing	4 (1.9)	4 (3.7)	0 (0.0)	0 (0.0)
**Ethnicity**
Hispanic	16 (7.7)	14 (12.8)	2 (2.9)	0 (0.0)
Non-Hispanic	185 (88.9)	93 (85.3)	64 (92.8)	28 (93.3)
Missing	7 (3.4)	2 (1.8)	3 (4.3)	2 (6.7)
**Age**
19–30 years	22 (10.6)	16 (14.7)	4 (5.8)	2 (6.7)
31–40 years	50 (24.0)	29 (26.6)	14 (20.3)	7 (23.3)
41–50 years	44 (21.2)	27 (24.8)	12 (17.4)	5 (16.7)
51–60 years	57 (27.4)	25 (22.9)	19 (27.5)	13 (43.3)
Over 60 years	33 (15.9)	11 (10.1)	19 (27.5)	3 (10.0)
Missing	2 (1.0)	1 (0.9)	1 (1.4)	0 (0.0)
**Undergraduate degree**				
None	6 (2.9)	6 (5.5)	0 (0.0)	0 (0.0)
Associate degree	17 (8.2)	11 (10.1)	6 (8.7)	0 (0.0)
Bachelor degree	144 (69.2)	68 (62.4)	49 (71.0)	27 (90.0)
Missing	41 (19.7)	24 (22.0)	14 (20.3)	3 (10.0)
**Certificates and advanced degrees**				
Certificate program	4 (1.9)	0 (0.0)	0 (0.0)	4 (13.3)
Masters degree	68 (32.7)	23 (21.1)	26 (37.7)	19 (63.3)
Doctorate degree	10 (4.8)	2 (1.8)	5 (7.2)	3 (10.0)
Professional degree	24 (11.5)	12 (11.0)	8 (11.6)	4 (13.3)

### Employment characteristics

Table 2 describes the employment characteristics of the 208 respondents who responded to this section of the survey. The majority of those who responded to the survey were employes of local health departments in Nebraska (*N* = 103/208; 49.5%). Almost a third of the respondents (*N* = 67/208; 32.2%) were employed by the State DHHS. Employes of tribal health departments, clinics, and American Indian related organizations accounted for 16.3% (*N* = 34/208) of the respondents. The remaining respondents were employed by other public health organizations in the state (*N* = 4/208; 1.9%). Over a third of the respondents (*N* = 73/208; 35.1%) did not disclose their full-time equivalent (FTE) status. Of those who did (*N* = 135/208; 64.9%), the majority was employed full time (*N* = 119/208; 88.1%). The respondents had worked in public health for an average of 13.9 years (SD = 11.2). The mean tenure in public health for respondents were 11.1 years for tier one, 16.5 years for tier two and 15.3 years for tier three.

**Table 2:** **Employment characteristics of respondents**.

	Total Respondents	Tier One	Tier Two	Tier Three
	*N* = 208	*N* = 109	*N* = 69	*N* = 3
	*N* (%)	*N* (%)	*N* (%)	*N* (%)
**Organization**
State Dept of Health and Human Services	67 (32.2)	22 (20.2)	30 (43.5)	15 (50.0)
Local Health Department	103 (49.5)	63 (57.8)	30 (43.5)	10 (33.3)
Tribal Health Departments, Clinics and Organizations	34 (16.3)	21 (19.3)	9 (13.0)	4 (13.3)
Other	4 (1.9)	3 (2.8)	0 (0.0)	1 (3.3)
**Job description**
Administrator/management	13 (6.3)	0 (0.0)	11 (15.9)	2 (6.7)
Communicable disease specialist/disease intervention specialist	4 (1.9)	4 (3.7)	0 (0.0)	0 (0.0)
Community health worker	3 (1.4)	3 (2.8)	0 (0.0)	0 (0.0)
Computer/technology	5 (2.4)	1 (0.9)	4 (5.8)	0 (0.0)
Dentist	1 (0.5)	0 (0.0)	1 (1.4)	0 (0.0)
Public health leader (e.g., Director, CEO, CFO, department head, bureau chief, etc.)	18 (8.7)	3 (2.8)	7 (10.1)	8 (26.7)
Emergency management	5 (2.4)	3 (2.8)	2 (2.9)	0 (0.0)
Epidemiologist	7 (3.4)	2 (1.8)		5 (16.7)
First responder (EMT, paramedic, fire, rescue, etc)	9 (4.3)	5 (4.6)	4 (5.8)	0 (0.0)
Health educator	2 (1.0)	1 (0.9)	1 (1.4)	0 (0.0)
Medical examiner/coroner/mortician	22 (10.6)	14 (12.8)	8 (11.6)	0 (0.0)
Nurse practitioner/physician assistant	26 (12.5)	18 (16.5)	8 (11.6)	0 (0.0)
Nutritionist/dietician	2 (1.0)	1 (0.9)	1 (1.4)	0 (0.0)
Pharmacist	9 (4.3)	8 (7.3)	1 (1.4)	0 (0.0)
Program coordinator/manager	26 (12.5)	9 (8.3)	10 (14.5)	7 (23.3)
Social worker/counselor	9 (4.3)	8 (7.3)	1 (1.4)	0 (0.0)
Administrative support staff	14 (6.7)	10 (9.2)	4 (5.8)	0 (0.0)
Administrative technical support staff (laboratory technician, X-ray technician, dental assistant, etc)	6 (2.9)	6 (5.5)	0 (0.0)	0 (0.0)
Other	21 (10.1)	13 (11.9)	6 (8.7)	2 (6.7)
Missing	6 (2.9)	0 (0.0)	0 (0.0)	6 (20.0)
**Full-time status**
Employed full time	119 (57.2)	57 (30.5)	43 (62.3)	19 (63.3)
Employed part time	16 (7.7)	14 (12.8)	2 (2.9)	0 (0.0)
Missing	73 (35.1)	38 (34.9)	24 (34.8)	11 (36.7)

	**Mean (SD)**	**Mean (SD)**	**Mean (SD)**	**Mean (SD)**

**Mean tenure in public health**	13.9 (11.2)	11.1 (10.4)	16.5 (12.2)	15.3 (8.6)

### Perceived level of importance

On a scale from 1 to 5 (1 = Not at all important; 5 = Very important), respondents were asked to rate the level of importance of each of the eight competency domains. When all the responses were aggregated, cultural competency skills and communications skills emerged as the two most important competency domains (mean score = 3.8), while public health sciences skills emerged as the least important domain (mean score = 3.3). All three tiers’ professionals ranked public health sciences skills as the least important competency domain. Tier one respondents ranked cultural competency skills as the most important public health competency domain (mean score = 3.7). Respondents of tiers two and three ranked communication skills as the most important competency domains (mean score = 4.0 and 4.1, respectively). Tier two’s respondents also perceived financial and management skills and leadership and systems thinking skills as very important competency domains (mean score = 4.0). Compared to tiers two and three, tier one respondents had lower perceived level of importance scores for each of the domains (3.1–3.7 vs. 3.5–4.1).

### Perceived level of proficiency

Respondents rated their self-perceived level of proficiency on each of the public health competencies, on a scale of 1 through 5 (1 = None; 5 = Expert). When all responses were aggregated, the mean proficiency score was below 3.0 (intermediate) for all eight competency domains. Using aggregate data from all respondents, the mean proficiency score was highest for communication skills (mean score = 2.9) and the lowest for policy development/program planning skills (mean score = 2.5). For all competency domains, respondents perceived their level of proficiency to be between the basic and intermediate levels.

Amongst tier one’s respondents, the mean proficiency scores were highest for cultural competency skills, communication skills, and community dimensions of practice skills (mean score = 2.6) and the lowest for policy development/program planning skills (mean score = 2.2). The mean proficiency score was highest for communication skills (mean score = 3.0) and the lowest for policy development/program planning skills (mean score = 2.6) amongst tier two’s respondents. Tier three’s respondents rated themselves as more proficient in community dimensions of practice skills and communication skills (mean score = 3.3) and least proficient in policy development/program planning skills (mean score = 2.9). In general, for each domain, the self-perceived proficiency score was highest for tier three, followed by tier two and tier one. The public health workforce professionals surveyed perceived that they were least proficient in the area of policy development and program planning and most proficient in communication skills.

### Need for training

The respondents’ need for training was determined based on calculated need scores.

Over 80% of respondents indicated that there was a need for training in all eight domains (Range = 81.1–91.7%). The three domains with the highest proportion of respondents with Need for Training scores of above 0 were financial planning and management skills (*N* = 111; 91.7%), community dimensions of practice skills (*N* = 110; 90.2%), and analytical/assessment skills (*N* = 116; 89.9%). The domains in which the lowest proportion of respondents had a Need for Training score of above 0 was cultural competency skills (*N* = 103; 81.1%) and Basic Public Health Science Skills (*N* = 104; 81.9%). The need for training scores were highest amongst tier two respondents across all domains (Table 3).

**Table 3:** **Proportion of respondents with a need for training across domains and tiers**.

Variable	Total responses		Tier one		Tier two		Tier three	
	Total	Need for training score 1 or above	Total	Need for training score 1 or above	Total	Need for training score 1 or above	Total	Need for training score 1 or above
	*N*	*N* (%)	*N*	*N* (%)	*N*	*N* (%)	*N*	*N* (%)
Financial planning and management	121	111 (91.7)	59	53 (89.8)	43	42 (97.7)	19	16 (84.2)
Community dimensions of practice	122	110 (90.2)	61	53 (86.9)	41	38 (92.7)	20	19 (95.0)
Analytical/assessment	129	116 (89.9)	66	58 (87.9)	44	42 (95.5)	19	16 (84.2)
Leadership and systems thinking	124	110 (88.7)	61	54 (88.5)	44	41 (93.2)	19	15 (79.0)
Communication	126	109 (86.5)	63	55 (87.3)	43	38 (88.4)	20	16 (80.0)
Policy development/program planning	121	104 (86.0)	60	51 (85.0)	43	39 (90.7)	18	14 (77.9)
Basic public health sciences	127	104 (81.9)	64	50 (78.1)	43	39 (90.7)	20	15 (75.0)
Cultural competency	127	103 (81.1)	65	54 (83.1)	42	36 (85.7)	20	13 (65.0)

### Perceived level of interest in training

Respondents were asked to rate their interest in training on a scale of 1–5 (1 = Not at all interested; 5 = Very interested). The mean level of interest in training ranged from 2.6 to 2.9 across all eight domains, indicating that respondents were slightly to moderately interested in training. Cultural competency skills and public health sciences skills emerged as the domains in which respondents had the highest (mean score = 2.9) and lowest (mean score = 2.6) levels of interest in training, respectively. In general, tier three’s respondents had the lowest level of interest in training (scores 2.2–2.6); while tier two’s respondents had the highest interest in training (scores 2.9–3.2) for all domains. Tier one’s respondents were most interested in training on cultural competency skills (mean score = 2.9) while respondents of tiers two and three were most interested in training on leadership and systems thinking skills (mean scores 3.2 and 2.6, respectively).

### Topical and skill areas of importance

Upon aggregation of all responses, obesity, health disparities, physical activity, chronic diseases, and diabetes ranked as the top five topical areas of importance to the respondents. Obesity and chronic diseases remained in the top five topical areas for all three tiers. Respondents identified program evaluation, team building, quality assurance, advocacy, and needs assessment as the five most important skill areas. The top five skill areas were similar across all three tiers.

### Technology access and training needs

Respondents also provided information on the various computer-related tasks they performed at work, as well as their perceived need for training in carrying out these tasks. Across all three tiers of respondents, a substantial proportion (43.5–47.3%) of respondents reported needing additional training in creating visual aids for presentation, developing a database, creating spreadsheets, conducting statistical analysis, and making web pages.

### Public health training preferences

The respondents preferred instructor-led training sessions. When we asked respondents to rank their preference for a number of training formats on a scale of 1 (no benefit) to 5 (great benefit), the three training formats that participants rated the highest were (a) on-site multi-day workshop with instructor, (b) regional state-wide training with instructor, and (c) interactive computer-based training with an instructor and other students, completed in a specific timeframe. All three tiers preferred these three formats. More than half (57.3%) of the participants, preferred training to be organized as a workshop, or conference, over several days (e.g., 2–3 days). A similar proportion of respondents (56.5%) preferred a distance education training option (i.e., via satellite, videoconferencing, or a web-based course).

Respondents, regardless of tier, were mostly motivated to pursue continuing education by the desire to gain a better understanding of their job-related area, to broaden their skill base, and to stay current in their career field. Interaction with instructors and other training participants was very important to respondents. Interestingly, while respondents in tiers one and two were most concerned about the opportunity to interact with others when participating in public health training, respondents in tier three were more concerned about flexibility. Tier three respondents cited the opportunity to start at any time and complete training at their own pace as the two most important factors in training.

We also asked respondents to select the training/education options participants indicated greatest interest in pursuing options in the next 3 years. The options ranged from short courses to PhD degree programs. When responses from all participants were aggregated, the majority of participants (55.7%) indicated interest in enrolling in short courses. Continuing education credits and certificate programs were also popular choices (43.5 and 32.1%, respectively). These three options were the most popular amongst all three tiers.

### Perceived barriers to training

The factors identified as potential barriers to training were consistent across tiers. The five most common barriers cited in all three tiers included cost, time/scheduling conflicts, distance/travel, workload, and family. Other mentioned factors included age, lack of motivation and encouragement, course availability, and the applicability of course contents to rural settings.

## Discussion

To our knowledge, the needs assessment conducted by the Great Plains PHTC was the first comprehensive public health workforce training needs assessment ever conducted in Nebraska. The study informed the content and format of programing offered and allowed the PHTC to provide training and education sessions targeting Nebraska’s public health workforce’s priority areas for learning and continuing education. Following the analysis of the needs assessment data, we conducted over 20 trainings that considered education and training priorities identified by survey participants. Tier one’s respondents, the most ethnically diverse group, were particularly interested in receiving training on cultural competency skills, while respondents of tiers two and three were most interested in training on leadership and systems thinking. As a result, we offered trainings on cultural competence and community-based participatory research with tribal communities that integrated cultural competence.

Although the public health workforce professionals surveyed perceived that they were least proficient in the area of policy development and program planning, the greatest needs for training were in financial planning and management skills and analytical/assessment skills across all tiers. We, therefore, offered the St. Louis University Evidence Based Public Health Course for local public health department practitioners. The course addressed areas in which survey participants indicated least proficiency and greatest need for training (15, 16).

Respondents identified obesity, health disparities, physical activity, chronic diseases, and diabetes as the top five public health topical areas. These topical areas align with state and national public health priorities and strategies for improving population health. The 2013 Nebraska state health improvement plan’s (SHIP) priority strategic issues include addressing associated risk factors such as obesity, physical activity, and diabetes to reduce heart disease (17). The national prevention strategy (NPS), called for by the 2010 Affordable Care act, has identified seven priority areas consistent with those identified in the results of Nebraska’s needs assessment (18). To promote the competence among Nebraska public health professionals in the five identified topical priority areas, in partnership with national, state, and local public health organizations, in 2012 we hosted two regional NPS Summits in Omaha and Grand Island. A total of 129 individuals attended the sessions of whom 95% agreed or strongly agreed that the sessions were useful in practice.

In general, the respondents preferred instructor-led interactive training sessions. In response to this finding, we conducted a number of face-to-face learning opportunities including the “leadership speaker series.” The speaker series provided the opportunity for practitioners to learn from influential public health leaders throughout the nation, including the president of the American Public Health Associations, and leaders in health disparities and health policy in the US Department of Health and Human Services and academia, respectively. The PHTC also supported scholarships for Tribal, local, and state public health practitioners by giving the opportunity to attend the Great Plains Public Health Leadership Institute, a year-long leadership development program, which engages participants in instructor-led interactive training in addition to group sessions. Additionally, the Office of Public Health Practice at the UNMC, College of Public Health also provided face-to-face organizational development opportunities for specific agencies that requested them. These sessions ranged from a 2-h session on presentation skills to a full-day workshop focused on team dysfunction. Leadership training also addressed tiers two and three survey respondents’ high perceived level of interest for training in this topic and systems thinking skills.

While respondents in tiers one and two were most concerned about the opportunity to interact with the instructor and other training participants, tier three respondents were more concerned about flexibility in training scheduling. We attribute tier three participants’ interest in training scheduling flexibility due to the leadership positions they occupy within their own organizations as senior managers and CEOs. Factors identified as potential barriers to training included cost, time/scheduling conflicts, distance/travel considerations, workload, and family commitments. The intent of PHTC funds includes addressing barriers related to flexibility in training schedule. The use of Moodle as the learning management system (LMS) ensured access to on-line training without the need to travel long distances and enabled the delivery of training modules at no cost to participants.

In addition to education and training opportunities, local and state health departments used the results in preparation for the national public health accreditation board (PHAB) accreditation. Local health departments were able to use the department level data to create department-specific workforce development plans. We also shared the results from the needs assessment with additional stakeholders, including the Nebraska State Health Improvement Plan Advisory Coalition. The results from our study helped inform the selection of the SHIP priority strategic issues.

## Limitations

There are a number of limitations to our study. The assessment was very lengthy and could have resulted in respondents skipping or not honestly answering the questions. Survey fatigue also represents a likely reason for a low response rate. Public health practitioners constantly receive requests for data from Universities, National Organizations, State Departments of Health, and granting agencies. Therefore, we re-administered the survey to those health departments not having at least 90% response rate. While the needs assessment resulted in data agreements and important collaborative partnerships with tribal health departments in the state, these relationships took time to establish which may have contributed to the low response rates from this constituency. Presently no standardized PHTC needs assessment instrument exists which makes training and educational priorities difficult to compare across PHTC sites. The absence of a standardized PHTC needs assessment instrument underscores the need for the establishment of this type of tool. This would save time and resources and allow for comparisons across the region and nation resulting in more time and resources for programing.

## Conclusion

The findings from our needs assessment and continued support from the PHTC, allowed for a) the delivery of training initiatives that met the needs of the public health agencies and personnel in the Nebraska and b) the provision of data to public health stakeholders across Nebraska to inform program and policy development. Addressing cost and timing, barriers to engaging in public health training and education, as identified by survey participants, we provided free trainings, and in addition to offering them in-person, we provided programing on-line, allowing for scheduling flexibility.

The 2014 reduction in PHTC funding and accompanying movement to establish regional PHTCs resulted in the closing of the Great Plains PHTC. The closing of the Great Plains PHTC has had a deleterious effect on the offering of public health training and education opportunities for public health professionals in Nebraska. The elimination of the dedicated funds to Nebraska perpetuates the existing cycle of forcing unprepared practitioners into positional leadership roles. Additionally, without these dedicated funds the tribal, local, and state health departments have difficulty in meeting the education and training gaps identified in their workforce development plans.

Finally, the NPS vision includes moving the US from a society focusing on sickness and disease to one based on prevention and wellness (18). To effectively carry out the vision articulated by the NPS at the local level requires the presence of public health infrastructure where public health agencies are capable of assessing and responding to community needs. Having local and capable public health agencies necessitates a trained public health workforce, underscoring the important function that PHTCs play toward promoting the public’s health and their need for continuous funding.

## Conflict of Interest Statement

The authors declare that the research was conducted in the absence of any commercial or financial relationships that could be construed as a potential conflict of interest.

## References

[1] Council of Linkages Between Academia and Public Health Practice. Core Competencies for Public Health Professionals. (2010). Available from: http://www.phf.org/resourcestools/Pages/Core_Public_Health_Competencies.aspx

[2] Institute of Medicine. Committee for the study of the future of public health. The Future of Public Health. Washington, DC: National Academy Press (1988). p. 153–9.

[3] Institute of Medicine. Committee on assuring the health of the public in the 21st century. The Future of the Public’s Health in the 21st Century. Washington, DC: National Academy Press (2003). p. 360–3.

[4] Healthy People 2020-Improving the Health of Americans. (2011). Available from: http://www.healthypeople.gov/2020/topicsobjectives2020/overview.aspx?topicid=35

[5] Rosenstock L Silver GB Helsing K Evashwick C Katz R Klag M Confronting the public health workforce crisis: ASPH statement on the public health workforce. Public Health Rep (2008) 123:395–8. 19006982 10.1177/003335490812300322 PMC2289968

[6] Association of State and Territorial Health Officials. 2007 State Public Health Workforce Survey Results. (2008). Available from: http://www.astho.org/Programs/Workforce-and-Leadership-Development/2007-State-Public-Health-Workforce-Survey-Results/

[7] National Association of County and City Health Officials. The Local Health Department: Finding from the 2008 National Profile of Local Health Departments. (2010). Available from: http://www.naccho.org/topics/infrastructure/profile/upload/NACCHO_WorkforceReport_FINAL.pdf

[8] Chen LW Roberts SM Lampman M Xu L Jacobson J Palm D. Nebraska’s Local Health Department Workforce: Findings from a 2008 Survey of Local Health department directors. University of Nebraska Medical Center College of Public Health, Department of Health Services Research (2010). Available from: http://www.learningace.com/doc/353738/8d62af61f7d8de7fc466bb41edd6c88b/pr2010-1_rwjf

[9] Potter M Fertman C Eggleston M Holtzhauer F Pearsol J A public health training center experience: professional continuing education at schools of public health. J Public Health Manag Pract (2008) 14(4):E10–6. 10.1097/01.PHH.0000324576.81942.b1 18552638

[10] Association of Schools and Programs of Public Health. Demonstrating Excellence in Practice-Based Research for Public Health. (2006). Available from: http://www.aspph.org/educate/models/demonstrating-excellence-in-practice-based-research-for-public-health/10.1177/003335490612100102 PMC1497790 16416689

[11] Emory PHTC, Georgia PHTC. Public Health Training Center Program in Georgia: Results of the Public Health Workforce Training Needs Assessment. (2011).

[12] Northwest Center for Public Health Practice. Oregon State Assessment Tool. (2012). Available from: http://www.nwcphp.org/evaluation/products-publications

[13] Scharff DP Andrews C Weimken T Kansas Local Health Department Workforce Needs Assessment. Heartland Centers for Public Health and Community Capacity Development (2006). Available from: http://www.kdheks.gov/olrh/download/2005_KS_Local_Needs_Assessment.pdf

[14] Ohio Public Health Training Center. Workforce Training Needs Opportunity. (2010). Available from: http://www.odh.ohio.gov/~/media/ODH/ASSETS/Files/lhd/osuworkforcetrainingneedsassessmentpresentation.ashx

[15] Brownson RC Baker EA Leet TL Gillespie KN True WR Evidence-Based Public Health. 2nd ed New York, NY: Oxford University Press (2011).

[16] Prevention Research Center in St. Louis. “Evidence-based Public Health Course.” Available from: http://prcstl.wustl.edu/training/Pages/Evidence-Based-Public-Health-Course.aspx

[17] Division of Public Health Nebraska Department of Health and Human Services. The Nebraska Public Health Improvement Plan: A Statewide Plan for Public Health Partners and Stakeholders to Improve the Health of Nebraskans. (2013). Available from: http://dhhs.ne.gov/publichealth/Documents/SHIP%20Plan.pdf

[18] National Prevention Council. National Prevention Strategy. Washington, DC: U.S. Department of Health and Human Services, Office of the Surgeon General (2011).

